# Epilepsy

**Published:** 1905-11

**Authors:** John W. Selman

**Affiliations:** Greehfleld, Ind.


### EPILEPSY.*

By DR. JOHN W. SELMAN, Greenfield, Ind.

Epilepsy is defined as a disorder of the nervous system, characterized by sudden convulsive seizures of temporary duration. The muscles of the parts affected being first in tonic spasm, then alternately contracted and relaxed; the attacks generally occurring at irregular intervals and being always accompanied by loss of consciousness, more or less complete in the typical disease. The movements also have no relation with those of ordinary life. In rare instances, however, one or more of these symptoms may be absent and yet the disease be epilepsy.

As far back as we have records of events the disease called epilepsy reaches. Long before medicine, as we know it at the present day, took the shape which separated it from witchcraft and sorcery. Civil writing incidently spoke of it, either describing the disease in detail or giving it a name which, in its meaning, described the affection. Long before the time of Galen and Hippo-

*Read before the Mississippi Valley Medical Association, Indianapolis, Ind., October 11, 1905.

crates we find mention of its character, and the famous Greek just mentioned has described it with a characteristic accuracy which seems as true of the disease to day as it was hundreds of years ago.

Almost every century since their time has borne in its medical annals some account of its symptoms, and probably no disease has ever given rise to more discussion, both medical and otherwise, than the one before us. Epilepsy by its constancy at all times and in all places fastened itself upon individuals and left accurate impressions on the minds of its observers. The very fact that epilepsy asserted itself in the bodies of its victims at the most inopportune times and before all men caused it to be brought to notice of the people more than all other affections even more widespread, but which by reason of their hidden nature were less frequently seen. Every form of explanation was attempted by the clergy. The laity and the most ignorant of the people shared the universal privilege of inventing new theories and therapeutic measures, and yet not one opinion has survived, and our building of knowledge of epilepsy contains no stone save those gathered in the last few years.

One of the first and most marked symptoms of an oncoming attack of epilepsy is a peculiar sensation felt in some portion of the body, generally below the brain, which gradually rises up over the patient, either rapidly or slowly, like an oncoming cloud, until the head having been reached the patient is immediately convulsed and unconscious, and almost instantly is seen to be in the very acme of the nervous storm. The duration of these tonic contractions rarely exceeds two minutes and in most cases is limited to but a few seconds. The excessive movements of the muscles of mastication force the increased quantities of liquid secreted by the salivary glands from the mouth into the form of froth, which is often stained with blood by reason of the injuries to the tongue. The spasms having ceased, the patient lies before us relaxed, unconscious and exhausted, and passes into a deep sleep or coma, which lasts a variable length of time and from which he or she can not be aroused, except very rarely and then with great difficulty. Even when the sleep has passed away the brain is evidently disturbed in its functions for several hours, or perhaps days, and headache is not rarely complained of after the patient seems like himself in other respects.

One of the most interesting and important of all symptoms is the so-called aura, and some difference of opinion has arisen as to the frequency of its occurrence, some authors stating it to be very rare, while others see it very constantly. There can be little doubt that in many cases it is as constantly present as in others it is absent, and it would appear that the nationality of the subject has something to do with the occurrence of this signal of the attack; at least if we may judge by the statements of the chief authors of each nation. In America the aura is wanting in a large proportion of the cases of true epilepsy. In England it occurs in about one-half the cases. In France and Belgium the aura appears to be present in more than half the cases in one form or another, as it is also in Germany, according to the most prominent neurologists.

The word aura is derived from the Latin signifying vapor, and its application to certain symptoms of epilepsy arises from the old Greek theory that the fit began by the ascent of a vapor in the veins of the extremeties. In later times it was imagined that the nervous impulse causing the spasm arose in the part where the aura first appeared since the attack could be put aside by the tightening of a ligature around the arm or leg, but this is held by most of the students of the disease at the present day to be impossible.

The circulation, the only changes are those brought about by the accompanying asphyxia. In some cases there is a stoppage of the pulse. This occurs when the inhabitory cardiac filaments of the vagus are affected. Every one agrees, of course, that during the violence of the muscular movements the force and rapidity of the circulation is increased, and particularly the arterial pressure. It is shown that during the clonic stage of the convulsion that arterial pressure is increased to a very great extent, as well as the pulse rate, but that during the first or tonic stage the pulse rate falls and the rhythm is so altered that a complete systole and diastole may occupy six times the normal period. Afterward the pulse passes to the normal or a condition of increased force and frequency.

So far only the regular symptoms of an attack have been given, and it must not be considered that all cases of epilepsy are so fully accompanied by a long train of constant signs as have been described. For it is evident that almost every "case is a law unto itself," and is only surrounded by an atmosphere which stamps it as epilepsy.

A very important question connected, not only with the prognosis of epilepsy, but also with its relation to medical jurisprudence lies in the influence which the disease may exercise on the mental condition of the epileptic. And while we know that some of the greatest men that ever lived were afflicted with epilepsy, in my opinion epilepsy does not necessarily involve any mental change. Great mental impairment exists in some cases, but this is the exception rather than the rule. Females suffer in mental vigor more commonly than males. The apprehension is more frequently preserved than lost. Ulterior mental changes are rare. Depression of spirits are common in males, rare in females, but that excitability or temper is found in both sexes.

With regard to the hereditary transmission of epilepsy, as indeed with regard to the causation of all diseases by supposed hereditary taint, it must be remembered that in as much as the large majority of cases owe this malady to other causes than inherited tendancy, a certain number of those whose parents exhibit a like affection to their own may have become morbid independently of any hereditary taint. It is well known that many of the children of epileptic parentage are free from the disease, and it is quite clear that many epileptics descended from epileptic stock may have been exposed to causes of the malady, which would of themselves have been held sufficient to induce the disease independently of any constitutional taint. In the largest and most correct sense of the word the etiology of epilepsy is advanced but little by the discovery of hereditary taint, the causation may be thus thrown backwards but it is not explained. Far too great an amount of importance has been attached to excessive venery or masturbation. It is very common to hear suspicions expressed upon this point. Much more common, I think, than to hear any such statement of facts as should prove that epilepsy and masturbation have any special character or frequency of relation to one another. The one is a tolerably prevalent disease; the other a very widely distributed vice. There are multitudes of epileptics with regard to whom no such suspicion could ever be entertained, and there are, it is to be feared, much larger multitudes of masturbaters who have never become epileptics. I speak of these two causes as they are considered by some physicians, and the laity in general, as the main causes of epilepsy. Especial pains should be taken to diagnose epilepsy from many of the reflex epileptic phenomena which occur in nervous children. These cases are those so frequently reported as being cured by trifling surgical operations such as circumcision, cutting of eye muscles, etc. Abnormal movements of the head, a nodding movement of the head, may be a form of epilepsy. In children it would be accompanied by a momentary loss of consciousness. In regard to hereditary cases, I desire to state that in the treatment of over six hundred cases of epilepsy I only had one case that I could in any way trace back. The mother of this girl, aged nine years, had convulsions when 12 years old. She would have one or two a week. The attacks were pronounced by the attending physician, epilepsy. When she menstruated at the age of 14 years the attacks stopped and she had no symptoms until one year ago. She is now 45 years old and the mother of four children, all vigorous and healthy except the youngest, a girl of 9 years old, who has had regular epileptic seizures for two years. The father and mother, who live in the north part of this State, started to bring the girl to Greenfield to see me; they arrived safely in Indianapolis, and while standing on the street corner waiting for the interurban car the girl had a seizure and in an instant the mother fell in a hard fit. She had an attack every day for eight days, but she is now to all appearances well; as is also the girl. This spring a gentleman and his wife were out riding when a street car frightened the horse, causing him to run away, and throwing the man and his wife out. The man was badly bruised but the woman escaped injury. In fact physically she had not been injured, but the shock was of such magnitude as to produce an indellible impression upon her nervous system, causing seizures resembling epilepsy, and so pronounced by several physicians and treated as such. This was a case of pure hystero epilepsy and soon yielded to proper treatment.

Every physician acknowledges that the great cause of epileptic convulsions is the sudden liberation or explosion of nerve force, which sweeps everything before it. It must be remembered that epilepsy and other diseases may exist hand in hand, and for this reason the prognosis and diagnosis are to be carefully formed and given. January 2d a man twenty-eight years old was brought to me. He was five feet ten inches tall and his weight was 110 pounds. He was having epileptic seizures five and six times a week. There was no warning, no aura. Like a flash he was in hard convulsions. The general appearance of this man indicated great anemiamalnutrition with diarrhea. I at once used the subcutaneous injections of the normal saline solution. With the bitter tonics and arsenic, and at the expiration of ten days, the change in his condition was wonderful, and in three months he returned home apparently well. The causes of epilepsy are "toxic reflex and traumatic." And I will not follow out the common custom of detailing remedies as useful or not useful, and it should be borne in mind that the treatment of epilepsy is as various as the disease is variable in its forms and phases, and should in nearly all cases revolve itself into three divisions consisting in the removal of any exciting cause, in the checking of the convulsive tendency already set up and in the prevention of any further attacks by suitable drugs or other measures of relief. The treatment is governed largely by the cause and is medicinal or operative, according to the etiological factors at work.

In idiopathic epilepsy medicinal means must be followed. While in a case resulting from traumatism the depressed bone abscess or tumor must be removed. In those due to reflex irritation the peripheral source of trouble must be sought out and relieved. For it is illustrative of the true birth of medicine that the treatment of epilepsy is rapidly passing from the cloud of ignorance into the bright light of modern science. For years it has passed among men as something too intangible to explain; too far beyond their power of treatment to yield to any one however skillful he might be, yet in the past few years more progress has taken place in our knowledge as to its entire course than in all preceding years, and it is now acknowledged that by proper treatment a large per cent. of cases ot epilepsy are curable, but each case of epilepsy is a study within itself and should be so treated.

Reference: H. A. Hare.

